# Psychological pathway to emotional exhaustion among nurses and midwives who provide perinatal bereavement care in China: a path analysis

**DOI:** 10.1186/s12888-024-05534-4

**Published:** 2024-02-01

**Authors:** Jialu Qian, Gaoyan Wu, Cecilia Jevitt, Shiwen Sun, Man Wang, Xiangyu Sun, Xiaoyan Yu

**Affiliations:** 1 Zhejiang University School of Medicine, Hangzhou, China; 2 Surgery Department of Thyroid and Breast, Huashan Hospital Fudan University, Shanghai, China; 3 Midwifery Division, Department of Family Medicine, Faculty of Medicine, University of British Columbia, Vancouver, Canada; 4 Department of Obstetrics, Women’s Hospital School of Medicine, Zhejiang University, Hangzhou, China 1st Xueshi Road, Zhejiang Province 310006

**Keywords:** Perinatal bereavement care, Organisational support, Secondary traumatic stress, Burnout, Path analysis, Cross-sectional

## Abstract

**Background:**

A lack of confidence in perinatal bereavement care (PBC) and the psychological trauma experienced by nurses and midwives during bereavement care leads to their strong need for sufficient organisational support. The current study intended to test a hypothesised model of the specific impact paths among organisational support, confidence in PBC, secondary traumatic stress, and emotional exhaustion among nurses and midwives.

**Methods:**

A descriptive, cross-sectional survey was conducted in sixteen maternity hospitals in Zhejiang Province, China, from August to October 2021. The sample (*n* = 779) consisted of obstetric nurses and midwives. A path analysis was used to test the relationships among study variables and assess model fit.

**Results:**

Organisational support directly and positively predicted confidence in PBC and demonstrated a direct, negative, and significant association with secondary traumatic stress and emotional exhaustion. Confidence in PBC had a positive direct effect on secondary traumatic stress and a positive indirect effect on emotional exhaustion via secondary traumatic stress. Secondary traumatic stress exhibited a significant, direct effect on emotional exhaustion.

**Conclusions:**

This study shows that nurses' and midwives' confidence in PBC and mental health were leadingly influenced by organisational support in perinatal bereavement practice. It is worth noting that higher confidence in PBC may lead to more serious psychological trauma symptoms in nurses and midwives. Secondary traumatic stress plays an essential role in contributing to emotional exhaustion. The findings suggest that support from organisations and self-care interventions were required to improve confidence in PBC and reduce negative psychological outcomes among those providing PBC. The development of objective measures for assessing competence in PBC and organizational support are essential.

## Background

Perinatal bereavement refers to the loss experience of parents in the aftermath of a miscarriage, stillbirth, or neonatal death [1, 2], which may result in various psychological problems for some parents [3, 4]. Given that the psychological outcomes of parents are influenced by the ability of nurses and midwives to provide effective bereavement support [5, 6], those professionals should be fully equipped to provide perinatal bereavement care (PBC).

However, the reality is that nurses and midwives lack confidence in PBC, because they do not know how to provide bereavement care to parents [7, 8]. Moreover, indirect exposure to traumatic perinatal experiences through the provision of care can lead to adverse psychological responses among those professionals [9, 10]. In several studies, nurses and midwives reported a heavy emotional burden in providing PBC [11–13]. An investigation of 473 certified nurse-midwives showed that 29% reported high to severe secondary traumatic stress [14]. Reduced confidence levels and high emotional exhaustion were reported following such traumatic experiences [9, 15].

Confidence is defined as “a feeling or belief that one will act in a right, proper, or effective way” [16]. Confidence aligns with the scope of self-efficacy, which refers to the belief of being able to accomplish a task or achieve a specific goal [17]. In this study, confidence in PBC refers to the confidence to provide bereavement services to grieving parents who experienced a perinatal loss. Confidence in PBC were conceptualised as bereavement support knowledge and skills [18].

Organisational support is a reflection of the organisation's concern for employee welfare and value for the employee's contribution. It plays an important role in establishing positive relationships with employees and motivating them to do their best [19]. Organisational support for well-being is the extent to which an organisation provides the resources, communication, reinforcement, and encouragement to enable employees to improve well-being [20]. In this study, organisational support referes to individuals' perceived levels and perceptions of support from ogranisation (clear policy, relaxing working environment, adequate staffing, available training, debriefing opportunities and so on) and workload in the context of PBC. The organisational response is essential to create the conditions and formal structures that promote support and enable health care professionals to provide high-quality PBC [5, 21, 22]. Organisational support in providing education and training showed benefits in developing confidence in PBC [23–25].

Secondary traumatic stress and emotional exhaustion are commonly seen negative psychological consequences among nurses and midwives in the context of PBC [25–27]. Secondary traumatic stress describes a condition that can occur for health professionals indirectly experiencing the trauma by hearing of or witnessing a traumatic event experienced by others [28]. The symptoms of secondary traumatic stress include intrusion (recurrent and intrusive distressing recollections of patients, including images, thoughts, or perceptions), avoidance (the avoidance of stimuli associated with the care of patients and the numbing of general responsiveness), and arousal (symptoms such as irritability, hypervigilance, difficulty concentrating) [29]. Emotional exhaustion is identified by feelings of fatigue, frustration, irritability and feeling worn out, depleting the employee's psychological resources [30]. The core dimension of burnout is emotional exhaustion which results from an extreme workload or personal work strain [31]. It has been reported that secondary traumatic stress is positively correlated with emotional exhaustion [29, 31]. Secondary traumatic stress is a predictor of emotional exhaustion [31, 32].

Organisational support has important effects in decreasing secondary traumatic stress and emotional exhaustion [15, 33]. Organisational efforts such as increased staffing and material support, lower workload and a greater perceived person-job congruence have demonstrated benefits in relieving secondary traumatic stress [34, 35] and emotional exhaustion [36–38]. In addition to the support from the literature cited, the relationships between organisational support, secondary traumatic stress and emotional exhaustion were also supported by a theoretical model: the job demands-resources model [39]. The model proposed that high job demands may result in workers experiencing burnout and mental illnesses. Job resources could minimise the related psychological costs. In line with the theory, nurses and midwives may experience secondary traumatic stress and emotional exhaustion because the provision of PBC requires additional psychological exertion or abilities. Organisational support, a kind of job resource, can decrease psychological costs, including secondary traumatic stress and emotional exhaustion.

Research regarding confidence in PBC is limited. We hypothesized that the path between confidence in PBC, secondary traumatic stress and emotional exhaustion was based on the summary of relationships among self-efficacy, secondary traumatic stress and emotional exhaustion. Confidence in PBC was considered to belong within the scope of self-efficacy in our study. Previous studies indicated that self-efficacy could negatively predict trauma-related stress [40, 41] and emotional exhaustion [41–44]. Hence, we deduced that confidence in PBC has negative relationships with secondary traumatic stress and emotional exhaustion.

Improving the quality of PBC services is a recognised global priority and an important component of primary health care [45]. It is essential to build a professionally competent and mentally healthy team of nurses and midwives to provide the best PBC [46]. In recent years, studies of confidence in PBC and psychological problems among nurses and midwives in perinatal bereavement practice have accumulated [9, 22, 23, 47]. However, there is still insufficient information related to the relationships between variables concerning organisational support, confidence in PBC, secondary traumatic stress and emotional exhaustion among nurses and midwives. These variables have not yet been tested in one model.

**Fig. 1:**
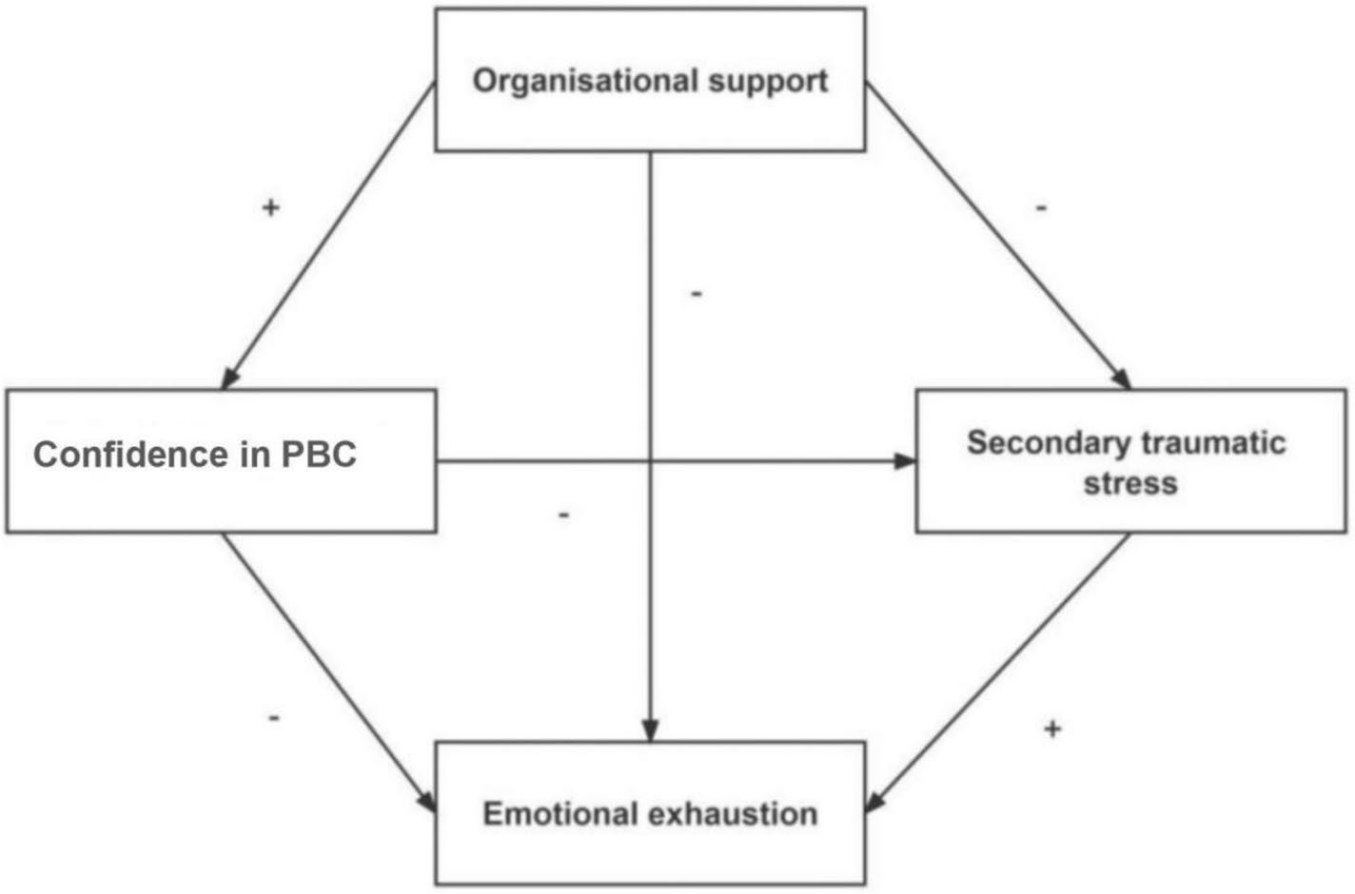
The hypothesized model

Thus, we aimed to examine a model of relationships between organisational support, confidence in PBC, secondary traumatic stress and emotional exhaustion in nurses and midwives and then reveal the direct and indirect paths through a path analysis. The hypothesised model is displayed in Fig. 1. We hypothesized the following: (1) Organisational support would show both a direct relationship with emotional exhaustion and an indirect relationship with emotional exhaustion through confidence in PBC and secondary traumatic stress; (2) organisational support not only has a direct effect on secondary traumatic stress but also has an indirect effect on secondary traumatic stress by confidence in PBC; and (3) confidence in PBC and secondary traumatic stress would each show both a direct relationship with emotional exhaustion and an indirect effect on emotional exhaustion through secondary traumatic stress.

## Methods

### Study design

A descriptive, cross-sectional study was conducted. This study received ethics committee approval (IRB no. 20210091). All methods were performed in accordance with the Declaration of Helsinki. The results of the study were reported following the guidance from the Strengthening the Reporting of Observational Studies in Epidemiology (STROBE) statement [48].

### Study participants

A convenience sampling method was adopted, and the participants were recruited from sixteen maternity hospitals in Zhejiang Province, China, between August and October 2021. To be eligible for participation in the study, individuals had to meet the following criteria: (1) they must have been midwives or nurses working in the maternity ward or delivery room, (2) they must have had experience providing PBC, and (3) they must have provided informed consent. Nursing or midwifery students, as well as those currently interning in hospitals, were excluded from the study. It is generally known that 200–400 samples are appropriate in the structural equation. In this study, 921 nurses and midwives completed the survey. After checking the data, 142 participants were excluded due to invalid questionnaires, primarily characterized by overly regular answers, which were considered indicative of potential response bias or inattention [49]. As a result, data from 591 obstetrics nurses and 188 midwives were eligible for data analysis, giving a final response rate of 76%.

## Measures

### Socio-demographic characteristics

The baseline characteristics of participants were evaluated by the self-designed questionnaire, which consisted of age, current area of practice, educational level, marital status, having children, length of work experience, and training in PBC.

### Confidence in PBC and organisational support

The Perinatal Bereavement Care Confidence Scale, which has been translated into Mandarin and validated in China, was used to measure nurses’ and midwives’ confidence in PBC and identify the psychosocical factors that impact on their confidence to provide bereavement support to provide PBC [22, 50]. It was used to measure bereavement support knowledge (13 items), bereavement support skills (eight items), self-awareness (eight items) and organisational support (11 items). Confidence in PBC was measured as the levels of nurses' and midwives' bereavement support knowledge and skills [22]. Organisational support subscale was used to assesses individuals' perceived levels and perceptions of support from ogranisation. Each item was rated on a five-point scale (1 = Strongly Disagree; 2 = Disagree; 3 = Neither Agree nor Disagree; 4 = Agree; 5 = Strongly Agree). The total score of confidence in PBC ranged from 21 to 105 and organisational support ranged from 11 to 55. Higher scores correlate with greater confidence and perceived organsiational support. The Chinese version of Perinatal Bereavement Care Confidence Scale revealed good reliability, with Cronbach's alpha values ranging from 0.835–0.901 [50].

### Secondary traumatic stress

The Secondary Trauma Scale measures secondary traumatic stress. It is a subscale of the Professional Quality of Life Scale (ProQOL) [51]. It has been validated in China [52]. This subscale contains 10 items, each rated on a 5-point scale from 1 (not at all) to 5 (extremely). The total score ranged from 10 to 50, with a higher score representing more serious secondary traumatic stress symptoms. The Cronbach's alpha coefficient of the scale in this study was 0.81.

### Emotional exhaustion

The Chinese Burnout Inventory was adopted to measure burnout [53], with a total of 15 items and three dimensions. Emotional exhaustion is one of the major dimensions and consists of five items. Items were rated on a 7-point Likert response set from 0 (never) to 6 (every day), with higher scores indicating higher levels of emotional exhaustion. Total scores range from 0 to 30. The Cronbach's alpha coefficient for this subscale was 0.91.

### Data collection

The head nurses of selected departments were approached through formal electronic document informing them about the study's purpose and procedures. They issued questionnaires through the WeChat communications app to nurses and midwives who met the eligibility requirements. The head nurses were responsible for explaining the matters needing attention during the distribution process. A webpage containing the study objectives and instruments was hosted at www.wjx.cn. A link to the survey was automatically generated and then sent to eligible nurses and midwives who consented to participate. The online survey, which took approximately 20 min to complete, helped ensure that the submitted responses did not contain missing data because the questionnaires could be submitted only when all questions were answered. All participants signed electronic informed consent in the beginning of the questionnaires and they were guaranteed the confidentiality of private information.

### Data analysis

To ensure accuracy and validity, data were reviewed for errors and cleaned after data export. Descriptive statistics for the demographic characteristics of the participants and correlational analyses were calculated using IBM SPSS Version 27.0. The hypothesised model was tested by structural equation modeling-path analysis using IBM SPSS AMOS Version 27.0, which incorporated four variables: organisational support, confidence in PBC, secondary traumatic stress and emotional exhaustion. Path analysis is advantageous in identifying both direct and indirect effects. All analyses were two-tailed, and α was set at 0.05. Unstandardized (B) and standardized regression (β) coefficients, along with the standard errors and P-values for β, were used to calculate the direct and indirect effects among observed variables. In the current study, 5000 bootstrap samples with 95% bias-corrected confidence intervals were generated to gather certain estimates.

Several criteria tested the hypothesised model's fit, including the chi-square/degrees of freedom ratio (χ2/df) < 5 [54], a nonsignificant chi-square (χ2) value (*P* ≥ 0.05), the root mean square error approximation (RMSEA) < 0.080, standardised root mean square residual (SRMR) < 0.080, the comparative fit index (CFI) > 0.900 and the Tucker–Lewis index (TLI) > 0.900 [54, 55].

## Results

### Description of participants

Participants’ demographic characteristics are presented in Table 1. Our 779 participants’ mean age (standard deviation; SD) was 32.18 (6.78) years. Of the valid samples, 671 participants (86.1%) had a bachelor's degree, and 318 participants (40.8%) had a nurse practitioner job title. Most of our sample were married (66.1%) and had children (61.1%). Approximately 38.1% of participants had more than 10 years length of work experience. A total number of 685 (87.9%) reported not receiving PBC training.

**Table 1:** Demographic characteristics (*N* = 779)

Variables	*N* = 779	%
**Age** (years; mean = 32.18, SD = 6.78)		
**Occupation**		
Midwife	188	24.1
Obstetric nurse	591	75.9
**Education level**		
College degree or below	97	12.5
Bachelor’s degree	671	86.1
Master’s degree or above	11	1.4
**Marital status**		
Married	515	66.1
Unmarried	253	32.5
Divorced or widowed	11	1.4
**Have children**		
Yes	476	61.1
No	303	38.9
**Length of work experience**		
< 2 years	55	7.1
2 ~ 5 years	165	21.2
5 ~ 10 years	262	33.6
> 10 years	297	38.1
**Training in PBC**		
Yes	94	12.1
No	685	87.9

Table 2 shows Spearman correlations, scale means and standard deviations. The results showed that organisational support was positively correlated with confidence in PBC and negatively correlated with secondary traumatic stress and emotional exhaustion. Confidence in PBC was negatively correlated with emotional exhaustion. Secondary traumatic stress was positively correlated with emotional exhaustion.

**Table 2:** Spearman correlations, scale means and standard deviations (*N* = 779)

Variables	Mean	SD	1	2	3	4
Organisational support	33.81	6.47				
Confidence in PBC	67.06	10.74	0.669**			
Secondary traumatic stress	22.92	7.59	-0.083*	-0.004		
Emotional exhaustion	17.44	8.51	-0.245**	-0.143**	0.527**	

* *p* < 0.05

**p < 0.01

### Path analysis results

**Fig. 2:**
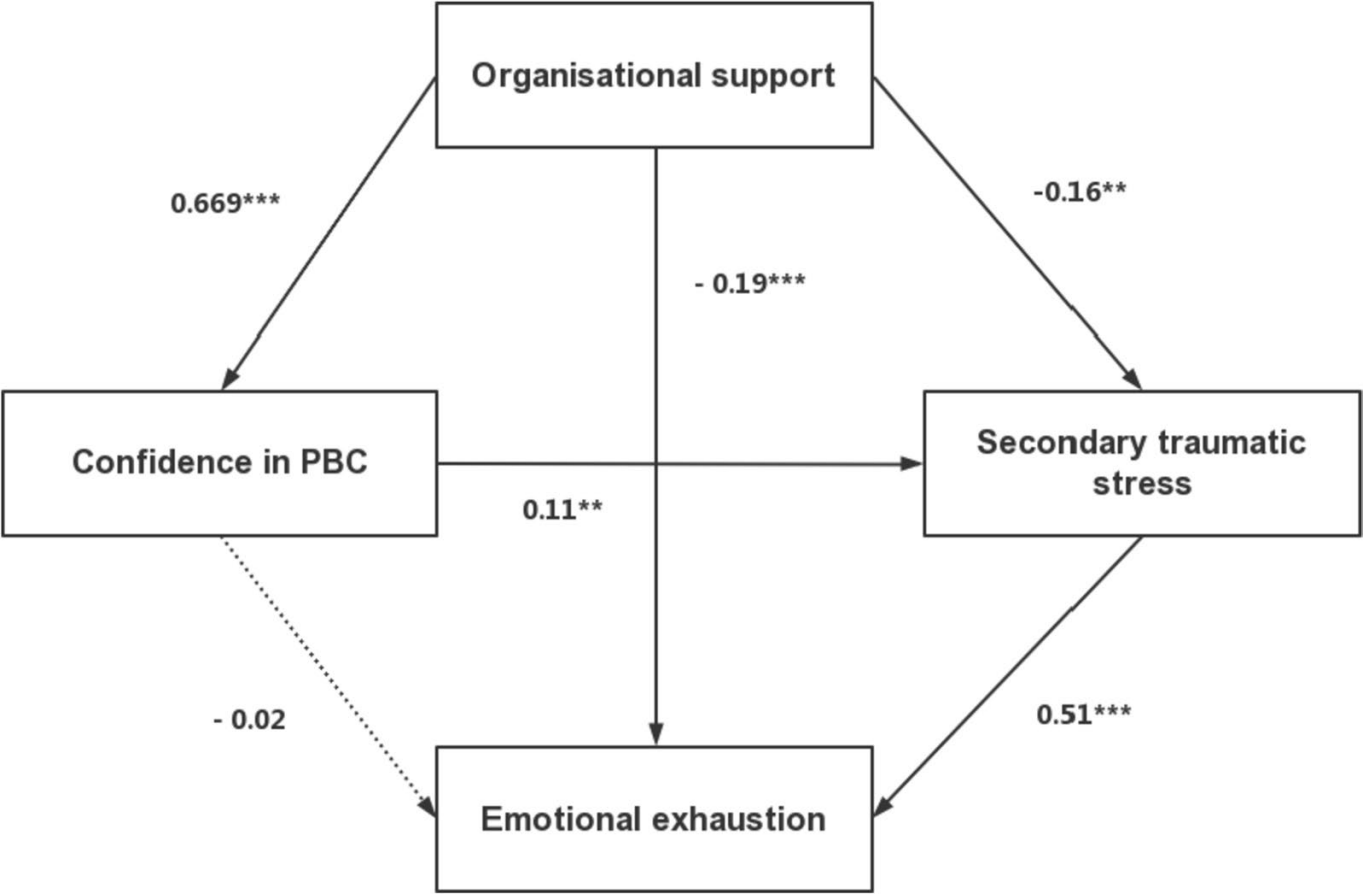
Final model for the whole sample (*N* = 779), with standardized beta weights and significant level

Figure 2 showed a good fit: χ2/df = 0.163 with a probability level of 0.687, RMSEA = 0.000, CFI = 1.000, TLI = 1.007, CFI = 1.000. All the fit indices confirmed that the data adequately fit our hypothetical path model. The parameter estimates of the paths between the variables in the final model are displayed in Table 3. The model is interpreted as follows. The results indicated that organisational support was directly predictive of confidence in PBC (β = 0.669, bootstrap 95% CI = 1.022, 1.195, *p* < 0.001), secondary traumatic stress (β = -0.155, bootstrap 95% CI = -0.288, -0.060, *p* = 0.001) and emotional exhaustion (β = -0.192, bootstrap 95% CI = -0.365, -0.146, *p* < 0.001). Confidence in PBC was associated with higher secondary traumatic stress (β = 0.108, bootstrap 95% CI = 0.001, 0.148, *P* = 0.024), and secondary traumatic stress was associated with higher emotional exhaustion (β = 0.511, bootstrap 95% CI = 0.500, 0.644, *p* < 0.001). Confidence in PBC exhibited an insignificant negative path on emotional exhaustion (β = -0.016, bootstrap 95% CI = -0.079, 0.050, *p* = 0.687).

**Table 3:** Standardized regression coefficients and standard errors for all pathways of the final model (*N* = 779)

Path		Final Model				
**Variable 1**	**Variable 2**	**B**	**SE**	**β**	**95% bias-corrected CI (1000 bootstraps)**	***P***
Organisational support	Confidence in PBC	1.112	0.044	0.669	1.022, 1.195	< .001
Organisational support	Secondary traumatic stress	-0.182	0.056	-0.155	-0.288, -0.060	0.001
Confidence in PBC	Secondary traumatic stress	0.076	0.034	0.108	0.001, 0.148	0.024
Confidence in PBC	Emotional exhaustion	-0.013	0.032	-0.016	-0.079, 0.050	0.687
Secondary traumatic stress	Emotional exhaustion	0.573	0.033	0.511	0.500, 0.644	< .001
Organisational support	Emotional exhaustion	-0.253	0.053	-0.192	-0.365, -0.146	< .001

*Abbreviation*: *PBC* Perinatal bereavement care

### Standardized direct, indirect, and total effects of all study variables

Table 4 lists each variable's standardized direct, indirect effects and total effects. As demonstrated in Table 4, organisational support to confidence in PBC demonstrated the strongest direct total effect (β = 0.669, bootstrap 95% CI = 0.621, 0.713, *P* = 0.002). Organisational support demonstrated both a direct (β = -0.155, bootstrap 95% CI = -0.252, -0.054, *P* = 0.004) and an indirect effect (β = 0.072, bootstrap 95% CI = 0.001, 0.142, *P* = 0.004) on secondary traumatic stress through confidence in PBC. There was only a direct pathway from organisational support to emotional exhaustion (β = -0.192, bootstrap 95% CI = -0.276, -0.111, *P* = 0.002). The indirect effect of organisational support on emotional exhaustion through the mediating roles of confidence in PBC and secondary traumatic stress was insignificant (β = -0.053, bootstrap 95% CI = -0.117, 0.007, *P* = 0.079). The model showed that confidence in PBC had no significant direct effect on emotional exhaustion (β = -0.016, bootstrap 95% CI = -0.099, 0.062, *P* = 0.687). Instead, its effect operated indirectly through confidence in PBC's effect on secondary traumatic stress (β = 0.108, bootstrap 95% CI = 0.001, 0.210, *P* = 0.044). There was only an indirect pathway from confidence in PBC to emotional exhaustion (β = 0.055, bootstrap 95% CI = 0.001, 0.107, *P* = 0.043). Secondary traumatic stress had only a direct effect on emotional exhaustion (β = 0.511, bootstrap 95% CI = 0.452, 0.567, *P* = 0.002).

**Table 4:** Standardised total, direct, and indirect effects of the variables

**Variable**	**Organisational support**	**P (BC)**	Confidence in PBC	**P (BC)**	**Secondary traumatic stress**	**P (BC)**
**Standardised total effects** **β (95% CI)**						
Confidence in PBC	0.669 (0.621, 0.713)	0.002	-		-	
Secondary traumatic stress	-0.083 (-0.151, -0.014)	0.018	0.108 (0.001, 0.210)	0.044	-	
Emotional exhaustion	-0.245 (-0.317, -0.176)	0.002	0.039 (-0.065, 0.128)	0.480	0.511 (0.452, 0.567)	0.002
**Standardised direct effects** **β (95% CI)**						
Confidence in PBC	0.669 (0.621, 0.713)	0.002	-		-	
Secondary traumatic stress	-0.155 (-0.252, -0.054)	0.004	0.108 (0.001, 0.210)	0.044	-	
Emotional exhaustion	-0.192 (-0.276, -0.111)	0.002	-0.016 (-0.099, 0.062)	0.708	0.511 (0.452, 0.567)	0.002
**Standardised indirect effects** **β (95% CI)**						
Confidence in PBC	-	-	-	-	-	-
Secondary traumatic stress	0.072 (0.001, 0.142)	0.040	-	-	-	-
Emotional exhaustion	-0.053 (-0.117, 0.007)	0.079	0.055 (0.001, 0.107)	0.043	-	-

*Abbreviation*: *BC* Bias-correctedm, *PBC* Perinatal bereavement care

## Discussion

To our knowledge, the present study is the first to statistically explore the associations between organisational support, confidence in PBC, secondary traumatic stress and emotional exhaustion among nurses and midwives by using path analysis, thereby explaining how these variables affected each other. Overall, the findings in this study suggest that the model has a good fit. The hypothetical model was tested, and the findings provided empirical support for some of the hypotheses. The study findings have implications for interventions to improve clinical perinatal bereavement practice and healthcare professionals' mental health in the context of PBC. The path coefficient from organisational support to confidence in PBC was 0.669, indicating the presence of an independent positive direct relationship for organisational support on confidence in PBC. This finding was supported by previous research where organisational support is necessary to create the conditions and formal structures that enable healthcare professionals to provide high-quality PBC [56]. Nurses and midwives expressed that there was a need for more organisational support in terms of debriefing opportunities and counseling services, and better organisation of patients' care [10, 22]. Relevant guidelines and principles emphasize the importance of strengthening organisational support to health care professionals by providing continuous training, thereby improving PBC [56–58]. Organisational support including developing evidence-based policies and protocols on key aspects of perinatal bereavement care, establishing multidisciplinary professional teams, strengthening training and providing emotional support to health care professionals could be referred to enhance the level of confidence in PBC among nurses and midwives for better bereavement care services [56].

Organisational support demonstrated a direct, negative, and significant association with secondary traumatic stress and emotional exhaustion. Similarly, the negative effects of organisational support on secondary traumatic stress [59] and emotional exhaustion were also observed in previous research [25, 60–62]. Organisational support was a protective factor of psychological well-being among healthcare workers [63, 64]. In this study, the results showed that secondary traumatic stress had a significant direct effect on emotional exhaustion. Previous research indicated that secondary traumatic stress is an important factor in developing emotional exhaustion [31, 32, 65]. Therefore, emotional exhaustion might be relieved by providing sufficient support and reducing secondary traumatic stress. A previous study reported that approximately 65% of nurses did not receive support from organisational leadership following traumatic incidents [66], which indicated that the existing organisational support was far from meeting their psychological needs. Organisations should strengthen support by carrying out self-care interventions, including mindfulness-based compassion training [67], resilience training [68] and peer support [66], to decrease secondary traumatic stress and emotional exhaustion among nurses and midwives.

As a means of reinforcing oraganisational support, strengthening training has become imperative. In regard to PBC training programs in China, only one recently established PBC training program has been implemented among nurses and midwives, and it has shown positive results [69]. Strengthening training as a means of enhancing organisational support for nurses and midwives. The training program not only focuses on enhancing confidence in PBC but also addresses the psychological well-being of nurses and midwives. More details can be found in the reference by Qian et al. [69]. This training program can serve as a reference for the development of PBC training both domestically and internationally. However, there is a need for further exploration and application of training in the field of PBC, especially in China, in the future.

The path analysis indicated that confidence in PBC had a positive direct effect on secondary traumatic stress and a positive indirect effect on emotional exhaustion in this path analysis, which were opposite to our hypothesis. On one hand, nurses and midwives with higher confidence in PBC scores need to pay more attention to the mood of the women who experience pregnancy loss, try to feel parents' emotional pain, have more empathy and provide better care for them. This process is accompanied by heavy emotional labor [12], which could negatively affect job satisfaction and well-being [70, 71]. If the nurses and midwives do not master emotional management skills, they may be easily affected by women's emotional grief and then experience increased secondary traumatic stress and emotional exhaustion [26, 27].

On the other hand, higher confidence in PBC scores do not mean that nurses and midwives adopt appropriate emotional labor strategies. There are three kinds of emotional labor strategies including surface acting, deep acting, and naturally felt emotions [72, 73]. A previous study found that surface acting involving the regulation of emotional expression and suppression of one's felt emotions was positively correlated with stress and burnout [74]. This may indicate that nurses and midwives with higher confidence in PBC may use the strategy of surface acting during PBC, which is similar to previous findings that nurses and midwives chose to keep silent, conceal emotions and not share feeling in the provision of PBC [10, 12]. Previous studies showed that using naturally felt emotions might facilitate emotional compatibility, offsetting burnout in nurses [75]. Even though nurses' and midwives' emotional regulation skills might be helpful for the achievement of organisational goals, administrators and managers need to realize that the natural expressions of nurses' and midwives' emotions in their interaction with patients could effectively reduce emotional exhaustion. It is vital to cultivate nursing and midwifery professionals' ability to spontaneously show their empathy and compassion instead of suppressing their emotions.

The present study supports the mediating role of confidence in PBC on the relationship between organisational support and secondary traumatic stress among nurses and midwives. In the path analysis, we found that organisational support had a negative direct effect on secondary traumatic stress, however, this effect was weakened by confidence in PBC. Therefore, this highlights that organisational support should not only focus on improving nurses' and midwives' professional ability to provide PBC, but also offer interventions that improve their ability to manage emotions during interactions with patients and encourage appropriate responses according to the patient context. This emotional approach might effectively foster their psychological well-being [74].

### Limitations

This study has several limitations. First, it used convenience sampling and was conducted in Zhejiang Province, which might constrain the generalisation of the results. Second, self-reported data may be influenced by social desirability bias, where participants could overestimate their confidence and perceived organizational support to align with expectations. Self-report measures potentially limit the accuracy of reflecting actual behavior. Although we took steps to minimize biases, including ensuring anonymity and emphasizing honesty, inherent subjectivity remains a limitation. Third, confidence in PBC, as an important indicator in this study, is distinct from competence; they are not the same concept. Our study primarily focuses on nurses' and midwives' confidence in their abilities to provide PBC, as self-assessment of confidence can influence behavior and actions. However, competence requires additional considerations, such as objective assessments of skills and actual behavior. Therefore, it is important to interpret the findings of this study with caution. Fourth, although the structural equation model has been widely adopted in examining causal relationships, there are still limitations to confirming causal relationships using cross-sectional data.

### Implications for future practice and research

First, the findings of this study have important implications for clinical health care managers to carry out professional PBC training and self-care interventions to improve confidence and enhance the psychological outcomes of nurses and midwives in the context of PBC. Second, considering confidence in PBC does not necessarily equate to competence and the limitation of self-report assessment, objective measures for assessing competence in PBC and organisational support need to be developed and applied. Further research to explore the relationship between confidence and competence is needed. Third, due to the limited research on PBC training in China, it is essential to develop and implement a systematic PBC training program in the future, taking into account the cultural and clinical practice characteristics of China.

## Conclusions

In sum, path analysis was first adopted in this cross-sectional survey of Chinese nurses and midwives to test the specific impact pathways among organisational support, confidence in PBC, secondary traumatic stress and emotional exhaustion. In the tested model, organisational support was an important psychosocial factor that impacts confidence in PBC, secondary traumatic stress and emotional exhaustion. Secondary traumatic stress plays an important role in contributing to emotional exhaustion. The findings that confidence in PBC leads to higher secondary traumatic stress and emotional exhaustion should be further verified.

## Data Availability

The data used to support the findings of this study are available from the corresponding author upon request.

## Abbreviations

PBC: Perinatal bereavement care
BC: Bias-corrected
CI: Confidence interval
DF: Degrees of freedom ratio
RMSEA: Root mean square error approximation
SRMR: Standardised root mean square residual
CFI: Comparative fit index
TLI: Tucker-Lewis index
SD: Standard deviation
STROBE: Strengthening the reporting of observational studies in epidemiology
